# Vessel density in early-stage primary open angle glaucoma and pseudoexfoliation glaucoma: a comparative controlled optical coherence tomography angiography study

**DOI:** 10.5935/0004-2749.20210051

**Published:** 2021

**Authors:** Ismail Umut Onur, Ozge Pinar Akarsu Acar, Ercan Cavusoglu, Fadime Ulviye Yigit

**Affiliations:** 1 Ophthalmology Department, Bakirkoy Dr. Sadi Konuk Training and Research Hospital, Bakirkoy, Istanbul, Turkey

**Keywords:** Vessel density, Optic nerve, Glaucoma, open-angle, Exfoliation syndrome Glaucoma, Tomography, optical coherence, Densidade vascular, Nervo óptico, Glaucoma de ângulo aberto, Síndrome de exfoliação, Glaucoma, Tomografia de coerência óptica

## Abstract

**Purpose:**

This study aimed to compare the vessel density of the optic nerve head and radial peripapillary capillary in the eyes with early-stage primary open angle glaucoma and pseudoexfoliation glaucoma and control eyes.

**Methods:**

With visual field mean deviation scores >-6.0 dB, 54 eyes from 37 patients diagnosed with primary open angle glaucoma (n=18) and pseudoexfoliation glaucoma (n=18) and healthy controls (n=18) were enrolled in this cross-sectional observational study. Retrieved from optical coherence tomography angiography, vessel density for the optic nerve head and radial peripapillary capillary were analyzed according to the distribution of the data and appropriate tests. The diagnostic accuracy of vessel density parameters was also assessed.

**Results:**

The whole-image vessel density of the radial peripapillary capillary and inside-disc vessel density of the optic nerve head were significantly lower in eyes with primary open angle glaucoma and pseudoexfoliation glaucoma compared to those in the control eyes (p<0.05). Compared to that in pseudoexfoliation glaucoma, the inside-disc vessel density of the optic nerve head was significantly lower in primary open angle glaucoma (p<0.05). Inferotemporal sector vessel density of the optic nerve head for both primary open angle glaucoma and pseudoexfoliation glaucoma was significantly lower than that of the controls (p=0.009). In discrimination of primary open angle glaucoma vs. control and pseudoexfoliation glaucoma vs. control, area under the receiver operating characteristic curve values for inside-disc vessel density of the optic nerve head were 0.855 and 0.731, respectively (p<0.001, p=0.018). However, in discrimination of primary open angle glaucoma vs. pseudoexfoliation glaucoma, area under the receiver operating characteristic curve values for whole-image and inside-disc vessel densities of the optic nerve head were 0.707 and 0.722 (p=0.034, p=0.023).

**Conclusions:**

Vessel densities of the optic nerve head and radial peripapillary capillary were significantly lower in eyes with primary open angle glaucoma and pseudoexfoliation glaucoma compared to healthy control eyes. In the early stage of glaucoma, the inside-disc vessel density of the optic nerve head slab may be lower in eyes with primary open angle glaucoma eyes compared to eyes with pseudoexfoliation glaucoma.

## Abstract (pt)

**Objetivo:**

Comparar a densidade vascular da cabeça do nervo óptico e a densidade capilar peripapilar radial em olhos em estágios iniciais de glaucoma primário de ângulo aberto e com glaucoma pseudoesfoliativo, bem como em olhos controle.

**Métodos:**

Este é um estudo observacional transversal, no qual foram incluídos 54 olhos com valores de desvio médio do campo visual superiores a -6,0 dB. Os olhos incluídos eram de 37 pacientes, diagnosticados com glaucoma primário de ângulo aberto (n=18), glaucoma pseudoesfoliativo (n=18) e controles saudáveis (n=18). Os valores de densidade vascular da cabeça do nervo óptico e a densidade capilar peripapilar radial foram obtidos a partir de angiografias por tomografia de coerência óptica, analisados de acordo com a distribuição dos dados e submetidos a testes estatísticos apropriados. Também foi avaliada a precisão diagnóstica dos parâmetros de densidade vascular.

**Resultados:**

Os valores para a densidade capilar peripapilar radial e no interior do disco óptico nas imagens inteiras foram significativamente menores no glaucoma primário de ângulo aberto e no glaucoma pseudoesfoliativo do que no grupo controle (p<0,05). A densidade vascular no interior do disco óptico na cabeça do nervo óptico foi significativamente menor no glaucoma primário de ângulo aberto do que no glaucoma pseudoesfoliativo (p<0,05). A densidade vascular no setor temporal inferior da cabeça do nervo óptico foi significativamente menor tanto no glaucoma primário de ângulo aberto quanto no glaucoma pseudoesfoliativo, em comparação com o grupo controle (p=0,009). A área abaixo da curva de ROC para a densidade vascular no interior do disco óptico na cabeça do nervo óptico, foi de 0,855 para a comparação do glaucoma primário de ângulo aberto com o controle (p<0,001) e de 0,731 para a comparação do glaucoma pseudoesfoliativo com o controle (p=0,018). Porém, na comparação do glaucoma primário de ângulo aberto com o glaucoma pseudoesfoliativo, os valores da área abaixo da curva de ROC para a densidade vascular na imagem inteira e no interior do disco óptico na cabeça do nervo óptico foram respectivamente de 0,707 e 0,722 (p=0,034, p=0,023).

**Conclusões:**

A densidade vascular na cabeça do nervo óptico e a densidade capilar peripapilar radial mostraram-se significativamente diminuídas no glaucoma primário de ângulo aberto e no glaucoma pseudoesfoliativo, em comparação com olhos controle saudáveis. Nos estágios iniciais do glaucoma, a densidade vascular no interior do disco óptico, na cabeça do nervo óptico, pode ser menor em olhos com glaucoma primário de ângulo aberto do que em olhos com glaucoma pseudoesfoliativo.

## INTRODUCTION

Glaucoma is a progressive optic neuropathy characterized by degeneration of the optic nerve head (ONH), retinal ganglion cell loss, and characteristic loss in the visual field (VF)^(1)^. Although increased intraocular pressure (IOP) is the most important factor for the progression of glaucoma^(2)^, there are additional potential risk factors, including reduced ocular perfusion^(3)^. Recent studies have shown that reduced ocular perfusion and microcirculatory deficiency in and around the ONH are associated with the development of glaucomatous optic neuropathy^(4)^.

One cause of glaucoma is pseudoexfoliation syndrome (XFS), a systemic disorder characterized by progressive accumulation of extracellular material in various tissues. In the eye, this accumulation causes obstruction of the trabecular meshwork and leads to an increase in IOP and development of pseudoexfoliation glaucoma (XFG)^(5)^. XFS also affects the ocular vasculature, including the ophthalmic artery, ciliary circulation, iris vessels, central retinal vein, and vortex veins^(6)^.

In addition to previous modalities, such as laser Doppler velocimetry, scanning laser Doppler flowmetry (LDF), laser speckle flowgraphy, color Doppler imaging, Doppler optical coherence tomography, and fluorescein angiography^(7-9)^, reproducible quantitative assessment of the microvasculature in the ONH, peripapillary retina (PPR), and macula can be performed noninvasively using optical coherence tomography angiography (OCT-A) with split spectrum amplitude-decorrelation angiography (SSADA)^(10-11)^. Recent studies have shown that the vessel density (VD) determined by OCT-A decreases in eyes with glaucoma^(12-15)^ and that reduction in VD is correlated with glaucoma severity^(16-17)^. However, this study aimed to use OCT-A to compare the VD of the optic disc and PPR in eyes with severity-matched early-stage primary open angle glaucoma (POAG) and XFG and healthy control eyes. Moreover, the diagnostic accuracy of the VD parameters for discriminating eyes with early-stage POAG and early-stage XFG and control eyes was also assessed.

## METHODS

This cross-sectional observational study was conducted at our tertiary eye clinic from July to September 2017 with approval from our Institutional Review Board (approval number, 2017/197). The study adhered to the tenets of the Declaration of Helsinki, and written informed consent was obtained from all individual participants included in the study.

POAG and XFG cases were identified from the glaucoma outpatient clinic record archives, and eligible patients were invited to participate in the study. Patients with POAG were included in the study if they had no history of any other ocular or systemic diseases causing VF damage, open angles on gonioscopy, characteristic glau comatous optic disc damage, thinning of the ganglion cell complex (GCC), or circumpapillary retinal nerve fiber layer (RNFL). Patients with XFG had clinically detected XFS in addition to POAG features. All patients with POAG and XFG were treated with topical antiglaucoma medication for IOP regulation.

Control subjects were selected from patients of the general ophthalmology clinic who had an IOP ≤21 mmHg, normal ONH parameters, intact neuroretinal rim, normal GCC and RNFL thickness, and normal standard automated perimetric parameters.

Patients with best corrected visual acuity (BCVA) <20/40, refractive error >+3.00 diopter (D) or <-6.00 D, history of intraocular surgery (apart from uncomplicated cataract surgery), any other ocular or systemic disorders (e.g., diabetic retinopathy) or neurological conditions that could cause VF loss or optic disc abnormalities, inability to perform reliably on automated VF testing, presence of any media opacities that could prevent good-quality OCT scans, and history of trauma to or inflammation in the eye were excluded.

VF analysis had been previously performed using the Humphrey Field Analyzer (Swedish Interactive Threshold Algorithm 24.2 test, Carl Zeiss Meditec, Dublin, CA). VF analyses were accepted as reliable if the false positive and false negative responses were <15%. Only eyes with early-stage POAG and XFG with VF mean deviation (MD) scores >-6.0 dB based on the Hoddap-Parrish-Anderson scale were included in our study.

The ONH rim area, optic disc area, cup-to-disc ratio, per ipapillary RNFL thickness, and macular GCC thickness measurements had been previously obtained using an RTVue-100 OCT device (Optovue Inc., Fremont, USA). We used the ONH map protocol to examine the RNFL parameters. This protocol creates an RNFL thickness map based on measurements around a circle 3.45 mm in diameter centered on the ONH. The protocol for the GCC scan was examining a square grid (7 × 7 mm) on the central macula after centering 1 mm temporal to the macula^(18)^. We accepted only high-quality images that had a signal strength index (SSI) >50. We used the average, superior, and inferior parameters for the mean GCC and RNFL measurements.

We obtained the OCT-A images of the ONH and PPR by conducting spectral domain (SD)-OCT on an RT XR Avanti device. We used the AngioVue software (Optovue, Inc., Fremont, CA, USA - prerelease version: 2016.1.0.23-beta), which employs the SSADA algorithm to detect the blood flow in an acquired volume. Each OCT-A volume contains 304 × 304 A scans, with two consecutive B scans, and the SSADA algorithm compares these B scans at the same location to detect blood flow using motion contrast^(10)^. The VD is described as the percentage area held by the large vessels and microvasculature in a specific zone.

**Figure 1:**
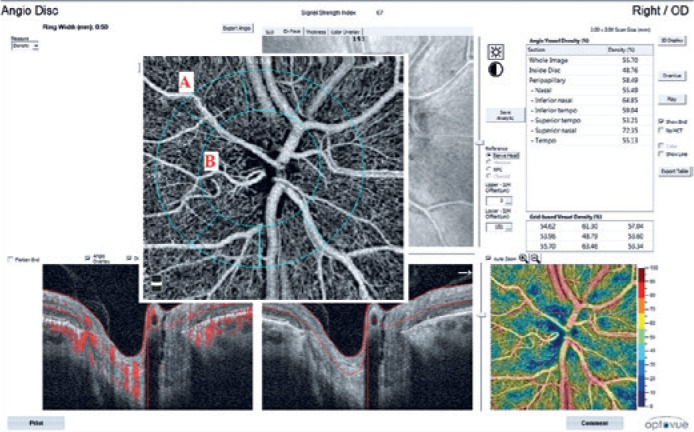
Optical coherence tomography angiography of the right optic nerve head of a patient with primary open angle glaucoma. The angiogram on the right shows the quantitative vessel density results of the optic nerve head. A, whole image; B, inside disc.

The optic disc scan covers an area of 3 × 3 mm, and the AngioVue software automatically fits an ellipse to the optic disc margin to calculate the average VD of the ONH. The PPR is described as a 0.70-mm-wide elliptical annulus extending from the optic disc boundary, and the average VD in this region is calculated by the software. The instrument divides the peripapillary region of both the ONH and radial peripapillary capillary (RPC) into six sectors according to the Garway-Heath Map. Then, it calculates the VD in each sector (nasal, inferonasal, superonasal, temporal, inferotemporal, and superotemporal sectors)^(19)^. In this study, we used the ONH VD results of the ONH angiogram calculated from the nerve head segment. This segment covers the region from the internal limiting membrane (ILM) to 151 microns below the ILM (Figure 1). We also referred to whole-image and peripapillary VD measurements from the angiography of the RPC segment that extends from the ILM to the posterior border of the RNFL.

### Statistical analysis

Statistical analysis was performed using the Statistical Package for Social Sciences (SPSS) version 22.0 (IBM Corporation, New York, USA). Descriptive statistics were presented as mean, standard deviation (SD) or median (Med), interquartile range (Q1-Q3), lowest value (Min), highest value (Max), frequency, and ratio (%), where appropriate. The normality distribution was evaluated for each parameter using the Kolmogorov-Smirnov test. Accordingly, parametric (for normal distribution) or nonparametric tests were applied. Parametric analysis of variance with post hoc Tukey test or nonparametric Kruskal-Wallis test with post hoc Mann-Whitney U test was used to analyze the independent variables. In case of non-normality, data were presented as Med and interquartile range. Chi-square test was used to analyze the qualitative data. A p-value <0.05 was considered statistically significant. The diagnostic accuracy of the ONH and RPC parameters for discriminating POAG and XFG (1), POAG and control (2), and XFG and control (3) was evaluated by area under the receiver operating characteristic curve statistics (AUROCs). The whole-image and inside-disc image VDs for both ONH and RPC parameters (slabs) were used for this purpose. The intraclass correlation coefficient was used to check for inter-eye correlation. A p-value <0.05 was considered statistically significant.

### Sample size

Referring to data in a previous study by Suwan et al.^(20)^ reporting significant difference in mean peripapillary capillary VD between XFG and POAG (29.4 ± 7.5 vs. 34.4 ± 6.6, p<0.05) and assuming an α-error of 0.05% and power of 80%, the required sample size was calculated to be 18 eyes for the standard effect size of 0.93.

## RESULTS

This cross-sectional observational study included 37 patients (15 women, 22 men) and a total of 54 eyes. The demographic and clinical features of the patients are summarized in Table 1. The number of patients with diabetes mellitus and hypertension was comparable between the POAG and XFG groups. All patients with POAG and XFG were treated with similar topical antiglaucoma medications. The IOP measurements were significantly lower in the POAG and XFG groups than in the control group (p=0.012 and p=0.001, respectively).

**Table 1:** Demographic and clinical characteristics of the study groups

	POZAG (n=18) (from 13 subjects)					XFG (n=18) (from 14 subjects)					Control (n = 18) (from 10 subjects)					P	
	Mean ± SD (n-%)		Med (Q1-Q3)			Mean ± SD (n-%)		Med (Q1 - Q3)			Mean ± SD (n-%)		Med (Q1-Q3)				
Age (year)			67	64	- 73			64	59 -	67			64	60	- 68	0.054	K
HT (number of patients)	2	15.4%				2	14.2%				1	10%					
DM (number of patients)	1	7.7%				1	7.1 %					-					
Sex Female Male	5 8	38.5% 61.5%				6 8	42.9% 57.1%				4 6	40% 60%					
Eye Right Left	10 8	55.6% 44.4%				12 6	66.7% 33.3%				9 9	50% 50%				0.589	X^2^
BCVA (logMAR)			0.1	0.0	- 0.2			0.0	0.0 -	0.1			0.0	0.0	- 0.1	0.142	K
1OP (mmHg)			15	13	- 17			14	13 -	16			19	17	- 19	**0.001**	K
Rim area (mm^2^)	1.11 ± 0.44						1.24 ± 0.48					1.45 ± 0.43				0.086	A
CCT (pm)	552 ± 35						530 ± 37					554 ± 44				0.134	A
Optic disc area (mm^2^)			2.3	2.0	-2.5			2.2	2.0-	2.5			2.0	1.9	- 2.3	0.259	K
C/D			0.7	0.6	- 0.8			0.7	0.6-	0.8			0.6	0.4	- 0.6	0.052	K

K = Kruskal-Wallis test (Mann-Whitney U test );

A = analysis of variance (Tukey test);

X ^^2^^= chi-square test; POAG= primary open angle glaucoma; XFG, pseudoexfoliation glaucoma; Q1= first quartile; Q3= third quartile; Med= median.

SD= standard deviation; BCVA= best corrected visual acuity; CCT= central corneal thickness; C/D= cup-disc ratio; IOP= intraocular Pressure; HT= hypertension; DM= diabetes mellitus.

Table 2 shows the GCC and RNFL examination results for all groups. All GCC parameters (average, superior, and inferior) were significantly lower in the POAG and XFG eyes than in the control eyes (p<0.05). Similar to the GCC results, all RNFL parameters (average, superior, and inferior) were significantly lower in the POAG and XFG groups than in the control group (p<0.05). No significant difference was noted for either the GCC or RNFL parameters between the POAG and XFG groups (p>0.05).

**Table 2:** Ganglion cell complex and retinal nerve fiber layer thickness measurements of the study groups

OCT angiography OCT	POAG (n=18) (from 13 subjects)			XFG (n = 18) (from 14 subjects)			Control (n = 18) (from 10 subjects)			P^K^	P^*^ POAG vs. XFG	P^*^ POAG vs. control	P^*^ XFG vs. control
	Median	QI	- Q3	Median	Q1 -	Q3	Median	Q1	- Q3				
GCC													
Average	90.5	82.2	- 97.9	88.4	82.3 -	91.8	96.7	94.0	- 99.6	**0.001**	**0.311**	**0.019**	**<0.001**
Superior	89.9	82.7	- 95.9	90.1	85.4 -	92.4	95.8	92.7	- 100.2	0.006	0.740	0.016	0.002
Inferior	91.2	81.4	- 97.7	87.3	78.3 -	92.4	97.8	94.5	- 99.9	0.001	0.217	0.023	<0.001
RNFL													
Average	89.2	85.0	- 92.1	84.6	79.3 -	95.0	102.4	96.3	- 106.2	<0.001	0.681	<0.001	<0.001
Superior	93.1	85.0	- 94.5	87.6	81.4 -	96.0	101.3	92.6	- 109.9	0.003	0.429	0.011	0.002
Inferior	86.7	82.0	- 94.1	85.2	75.6 -	95.8	100.8	95.3	- 106.7	<0.001	0.591	0.001	0.001
VF													
MD	-2.63	-4.48	- -1.62	-2.81	-4.12 -	-1.77	0.29	0.17	- 0.35	<0.001	0.937	<0.001	<0.001
PSD	3.32	1.82	- 3.69	2.91	2.06 -	3.36	1.46	1.42	- 1.76	<0.001	0.764	<0.001	<0.001

OCT= optical coherence tomography; POAG, primary open angle glaucoma; XFG= pseudoexfoliation glaucoma; Q1= first quartile; Q3= third quartile.

GCC= ganglion cell complex; RNFL= retinal nerve fiber layer; VF= visual field; MD= mean deviation; PSD, pattern standard deviation.

K P-values are based on Kruskal-Wallis test (^*^Mann-Whitney U test) (controlled by intraclass correlation coefficient for inter-eye correlation).

Table 2 also shows the VF parameters of the groups. The MDs were significantly lower in patients with POAG and XFG than in the control subjects (p<0.05), while the pattern SDs (PSD) were significantly higher in the POAG and XFG groups than in the control group (p<0.05). Neither the MD nor the PSD results showed a significant difference between the POAG and XFG groups (p>0.05).

Table 3 shows the results for the VD measurements of the ONH. The inside-disc image and inferotemporal sector VDs were significantly different between the groups (p<0.05). Post hoc analysis (Mann-Whitney U test) revealed that the VDs for the inside-disc and inferotemporal sectors were significantly lower in the POAG and XFG groups than in the healthy control group. In addition, the inside-disc VDs of the ONH were significantly lower in patients with POAG than in patients with XFG. However, no significant difference in inferotemporal sector VD between patients with POAG and XFG was noted. These ONH VD parameters did not show statistically significant inter-eye correlations (p<0.05, intraclass correlation coefficient).

**Table 3:** Optic nerve head vessel density measurements of the study group

OCT angiography %	POAG (n = 18) (from 13 subjects)			XFG (n=18) (from 14 subjects)			Control (n=18) (from 10 subjects)			pK	P^*^ POAG vs. XFG	P^*^ POAG vs. control	P^*^ XFG vs. control
	Median	Q1 -	Q3	Median	Q1 -	Q3	Median	Q1	- Q3				
ONH Inside	48.7	43.2 -	51.9	51.9	50.9 -	54.1	56.7	53.0	- 58.2	**<0.001**	**0.023**	**<0.001**	**0.018**
-Nasal	59.1	51.2 -	63.7	59.7	56.2 -	62.5	62.7	59.4	- 65.8	0.085			
-Inferonasal	61.2	58.4 -	63.1	63.1	59.2 -	65.1	64.0	59.4	- 66.7	0.052			
-Inferotemporal	59.2	54.7 -	66.0	59.5	58.1 -	65.7	67.8	64.9	- 70.1	0.009	0.856	**0.007**	**0.011**
-Superotemporal	59.9	52.4 -	67.1	64.1	58.2 -	67.8	65.5	63.0	- 68.8	0.103			
-Superonasal	58.0	53.0 -	62.3	62.0	58.6 -	64.6	60.8	54.9	- 63.5	0.243			
-Temporal	56.4	49.8 -	62.1	59.2	55.9 -	60.7	62.7	61.4	- 65.1	0.053			

OCT= optical coherence tomography; ONH= optic nerve head; POAG= primary open angle glaucoma; XFG= pseudoexfoliation glaucoma; Q1= first quartile; Q3= third quartile

K P-values are based on the Kruskal-Wallis test (^*^Mann-Whitney U test) (controlled by intraclass correlation coefficient for inter-eye correlation).

Table 4 shows the RPC VD results. The whole-image RPC VDs were significantly different between the groups (p<0.05), while no difference was found in RPC VDs for peripapillary sectors (p>0.05). Post hoc analysis (Mann-Whitney U test) showed that whole-image RPC VDs were significantly lower in the POAG and XFG groups than in the healthy control group (p<0.05). However, no statistically significant difference was detected in the whole-image RPC VD between the POAG and XFG groups (p=0.486). These RPC VD parameters did not also show statistically significant inter-eye correlation (p<0.05, intraclass correlation coefficient).

**Table 4:** Radial peripapillary capillary vessel density measurements of the study group

OCT angiography %	POAG (n=18) (from 13 subjects)			XFG (n = 18) (from 14 subjects)			Control (n=18) (from 10 subjects)			P^k^	P^*^ POAG vs. XFG	P^*^ POAG vs. control	P^*^ XFG vs. control
	Median	Q1 -	Q3	Median	Q1 -	Q3	Median	Q1	- Q3				
RPC Whole	53.4	50.8 -	54.7	54.0	52.3	56.1	57.1	54.8	- 58.7	*0.001*	0.486	*0.001*	*0.002*
-Nasal	60.8	54.3 -	64.4	61.6	56.8 -	64.3	61.5	58.4	- 65.0	0.724			
-Inferonasal	63.6	60.0 -	67.3	69.9	65.4 -	71.0	64.7	62.7	- 68.1	0.052			
-Inferotemporal	62.2	60.3 -	68.2	64.5	58.8 -	71.2	68.7	67.0	- 71.0	0.072			
-Superotemporal	63.2	58.6 -	69.4	70.7	61.3 -	74.6	69.7	63.7	- 71.9	0.052			
-Superonasal	60.9	57.6 -	63.5	66.0	59.0 -	68.7	61.6	55.5	- 64.5	0.057			
-Temporal	62.8	54.5 -	65.9	66.3	62.1 -	68.4	65.2	61.7	- 66.9	0.160			

OCT= optical coherence tomography; RPC, radial peripapillary capillary; POAG= primary open angle glaucoma; XFG= pseudoexfoliation glaucoma; Q1= first quartile; Q3= third quartile.

K P-values are based on the Kruskal-Wallis test (^*^Mann-Whitney U test) (controlled by intraclass correlation coefficient for inter-eye correlation).

**Figure 2:**
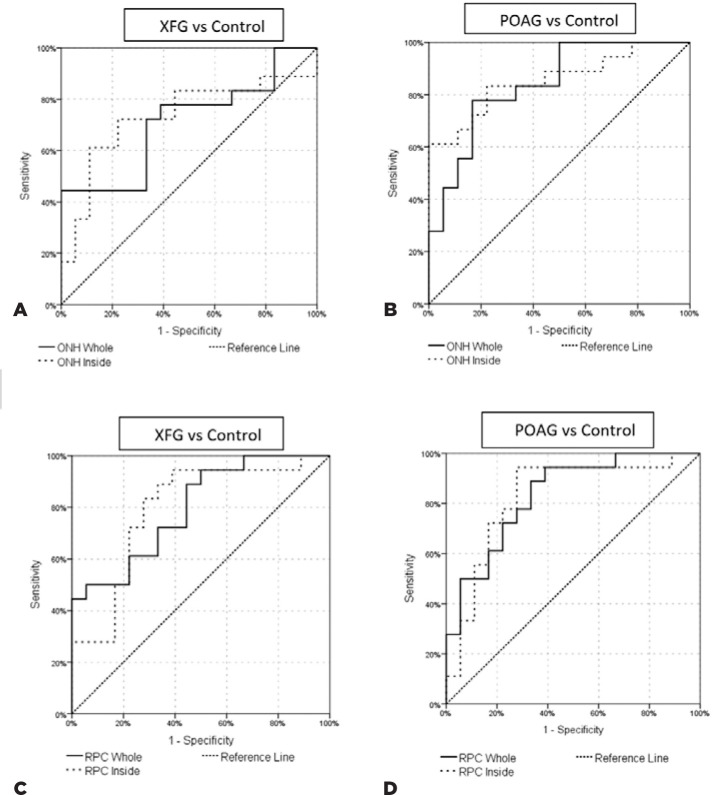
A) Area under the receiver operating characteristic curves (AUROCs) showing the diagnostic accuracy for discrimination between XFG vs control and POAG vs control using whole-image and inside-disc vessel density of ONH (A-B). B) Area under the receiver operating characteristic curves (AUROCs) showing the diagnostic accuracy for discrimination between XFG vs. control and POAG vs. control using RPC VD of the whole image and inside disc (C-D).

The diagnostic powers for discriminating between POAG and XFG, POAG and control, XFG and control were shown as AUROC values in table 5. Accordingly, both whole-image and inside-disc VDs of the ONH slab showed statistically significant AUROC values for discriminating POAG and XFG, whereas the RPC values for whole image or inside disc image did not reach statistical significance. However, for POAG vs. control and XFG vs. control discrimination, all ONH- and RPC-related whole-image and inside-disc VDs showed significantly higher AUROC values. Figure 2A and 2B shows the AUROCs of the POAG and XFG groups compared with the control group.

**Table 5:** Area under the receiver operating characteristic curve (AUROC) values of vascular parameters

		AUC	95% CI			P-value	
*POAG vs XFG* ONH whole		0.707	0.533 - 0.881			*0.034*	
ONH inside		0.722	0.551 - 0.893			*0.023*	
RPC whole		0.568	0.376 - 0.760			0.486	
RPC inside		0.503	0.309 - 0.697			0.975	
	*POAG vs control*						
	ONH whole	0.840	0.711	-	0.968	*0.001*	
	ONH inside	0.855	0.729	-	0.981	*0.000*	
	RPC whole	0.833	0.702	-	0.964	*0.001*	
	RPC inside	0.827	0.682	-	0.972	*0.001*	
*XFG vs control* ONH whole		0.710	0.537	-	0.883	*0.031*	
ONH inside		0.731	0.556	-	0.907	*0.018*	
RPC whole		0.796	0.653	-	0.939	*0.002*	
RPC inside		0.793	0.640	-	0.947	*0.003*	

AUC= area under the curve; POAG= primary open angle glaucoma; XFG= pseudoexfoliation glaucoma; ONH= optic nerve head; RPC= radial peripapillary capillary; CI= confidence interval.

## DISCUSSION

The ONH blood flow can be affected by the accumulation of pseudoexfoliation material, especially in the posterior ciliary arteries^(21)^. For example, Harju et al. used LDF to study blood flow in the lamina cribrosa region, rim area, and PPR in 50 patients with XFG or ocular hypertension with XFS in one eye and reported that advanced glaucomatous damage was associated with reduced flow in both the lamina cribrosa and rim area, but not in the PPR^(22)^. Conversely, Ocakoglu et al. reported that XFS was associated with reduced ocular blood flow in both the ONH and PPR in their study on 22 patients with XFS with the same device^(23)^.

Yuksel et al. used color Doppler imaging to investigate the orbital blood flow velocity in patients with XFG and POAG^(24)^. Despite no significant difference in the mean blood flow parameters between POAG and XFG, the authors reported that the blood flow velocities of the retrobulbar vessels were decreased in patients with XFG and POAG than in the control subjects. Martinez and Sanchez also used color Doppler imaging to study the hemodynamic parameters in the ophthalmic artery and short posterior ciliary arteries in patients with XFG and POAG^(25)^, and it showed lower blood flow in patients with POAG. One point that should be kept in mind is that previous studies mentioned above directly examined orbital blood flow velocity, whereas we measured retinal VD.

Suwan et al. were the first to use OCT-A ONH images (4.5 × 4.5 mm) to study 43 eyes with XFG and 31 eyes with POAG matched for VF MD to 33 eyes with XFS and 45 control eyes. They reported a significant decrease in VD of the RPC in eyes with XFG compared to eyes with POAG and eyes with XFS compared to control eyes^(20)^. They also mentioned, as a limitation of their study, that a relatively high proportion of older patients were included in the XFS and XFG groups and that advanced age could affect the microvasculature around the optic disc. However, another study by Park et al.^(26)^ compared the peripapillary RPC VDs (4.5 × 4.5) in age matched 39 eyes with XFG and 39 eyes ewith POAG using a swept-source OCT-A device and reported lower mean VDs in all sectors (significant in some) of the XFG and POAG groups. Similarly, Rebolleda et al. used the same OCT device (AngioVue) but 4.5 × 4.5-mm scans and also reported significantly lower mean peripapillary VD of the ONH in patients with XFG than in those with POAG^(27)^. Although we found a significant decrease in the whole-image VD of the RPC in the eyes with XFG and POAG compared to control eyes, we did not find a significant difference in the peripapillary sector VD of the RPC between patients with POAG and XFG as mentioned above. We conversely found a significantly lower peripapillary VD of the ONH of inside-disc image in the POAG group compared to that in the XFG group. Although there was no significant difference in mean age, in our study, a high proportion of older patients were included in POAG group compared to that in the XFG group. The contrast between our results and those of aforementioned studies might result from disease severity discrepancies of the eyes included in the studies; that is, we only enrolled eyes with very early-stage disease. However, a recent study by Pradhan et al., which compared severity-matched 39 eyes with XFG and 39 eyes with POAG, found no significant difference in peripapillary VD (4.5 × 4.5) of the RPC in contradiction to previous studies^(28)^. Another factor that might explain the difference between the results of these studies and those of our study is the size and resolution of the area captured on OCT-A, which was 3 × 3 mm (higher definition) in our study. Of note, distribution of optic nerve disc size (disc area) of the eyes in our study was appropriate for 3 × 3 evaluation (Table 1).

Regarding diagnostic accuracy, previous studies showed high AUROC values for RPC VD or whole-image VD of the ONH, thereby allowing discrimination between healthy controls and eyes with glaucoma. With respect to peripapillary VD, Liu et al. reported AUROC value of 0.938 for discrimination of normal eyes and eyes with glaucoma, in which glaucoma subtypes were not mentioned^(12)^. However, Chen et al. found AUROC value of 0.93 for peripapillary whole-image VD in discriminating eyes with POAG and healthy eyes^(13)^. In discriminating eyes with early-stage glaucoma (MD > -6.0 dB) and normal eyes, whole-image and inside-disc VDs showed AUROC values of 0.830 and 0.566, respectively, in the study by Chung et al.^(15)^. Similarly, these former and latter values were 0.740 and 0.756, respectively, in eyes with early-stage POAG and normal eyes by Kurysheva et al.^(29)^. Moreover, Chihara et al. showed AUROC value of 0.832 for the RPC VD in the POAG and control groups^(30)^, as whole-image and inside-disc VDs showed AUROC values of 0.90 and 0.73, respectively, in eyes with POAG and normal eyes in another study^(14)^. In the light of the above-mentioned results, our AUROC values were comparable within the range of the AUROC values reported for discriminating eyes with POAG and control eyes and even eyes with XFG and control eyes. Nevertheless, controversial results were reported in discriminating eyes with POAG and XFG by Suwan et al., who reported no significant AUROC values for the VD parameters for XFG and POAG^(20)^, whereas Rebolleda et al. showed statistically significant AUROC value, such as 0.720, for whole-image VD^(27)^. In agreement with Rebolleda et al.’s study, our study showed significant AUROC values for whole-image and inside-disc VD of the ONH slab in discriminating XFG and POAG. Admittedly, the relevant AUROC values are quite low for a powerful diagnostic classifier.

Compared to previous studies and particularly to that of Suwan et al.^(20)^, a major strength of our study is our enrollment of only early-stage POAG and XFG cases. This may produce outcomes that differ from those obtained with moderate or severe cases and favor a better understanding of the early changes in microvasculature. Of note, patients in both the XFG and POAG groups in this study were using topical antiglaucoma medication so that the mean IOP results of both groups were significantly lower than those of the control groups.

This cross-sectional study also has a number of limitations. One is the limited number of patients, which may affect the power of our results. A prospective study that includes large groups of patients would provide more reliable results. The borderline significance values for RPC VD on sectors were low, which likely resulted from the suboptimal power of the study. Another limitation is that our use of the 3 × 3 scans, despite being better for imaging details, may have yielded suboptimal results when compared with 4.5 × 4.5 or 6 × 6 scans for assessments of the peripapillary zone.

Therefore, similar to previous studies, we demonstrated reduced average values for ONH and PPR VD using with quantitative OCT-A in patients with POAG and XFG compared to those in controls. However, the findings of lower VD in eyes with POAG compared to that in eyes with XFG may suggest that vascular insufficiency plays a more significant role in the pathogenesis of early POAG than XFG. We found OCT-A to be useful as a noninvasive method for the evaluation of microvasculature in and around the ONH. This study showed a reduction in the ONH and PPR VD in patients with early-stage glaucoma.
